# Masked pre-training of transformers for histology image analysis

**DOI:** 10.1016/j.jpi.2024.100386

**Published:** 2024-05-31

**Authors:** Shuai Jiang, Liesbeth Hondelink, Arief A. Suriawinata, Saeed Hassanpour

**Affiliations:** a Department of Biomedical Data Science, Geisel School of Medicine at Dartmouth, Hanover, NH 03755, USA; b Department of Pathology and Laboratory Medicine, Dartmouth-Hitchcock Medical Center, Lebanon, NH 03756, USA; c Department of Epidemiology, Geisel School of Medicine at Dartmouth and the Department of Computer Science, Dartmouth College, Hanover, NH 03755, USA

**Keywords:** Digital pathology, Deep learning, Self-supervised learning, Transformer

## Abstract

In digital pathology, whole-slide images (WSIs) are widely used for applications such as cancer diagnosis and prognosis prediction. Vision transformer (ViT) models have recently emerged as a promising method for encoding large regions of WSIs while preserving spatial relationships among patches. However, due to the large number of model parameters and limited labeled data, applying transformer models to WSIs remains challenging. In this study, we propose a pretext task to train the transformer model in a self-supervised manner. Our model, MaskHIT, uses the transformer output to reconstruct masked patches, measured by contrastive loss. We pre-trained MaskHIT model using over 7000 WSIs from TCGA and extensively evaluated its performance in multiple experiments, covering survival prediction, cancer subtype classification, and grade prediction tasks. Our experiments demonstrate that the pre-training procedure enables context-aware understanding of WSIs, facilitates the learning of representative histological features based on patch positions and visual patterns, and is essential for the ViT model to achieve optimal results on WSI-level tasks. The pre-trained MaskHIT surpasses various multiple instance learning approaches by 3% and 2% on survival prediction and cancer subtype classification tasks, and also outperforms recent state-of-the-art transformer-based methods. Finally, a comparison between the attention maps generated by the MaskHIT model with pathologist's annotations indicates that the model can accurately identify clinically relevant histological structures on the whole slide for each task.

## Introduction

Histopathological slides are widely used in clinical settings for cancer diagnosis, prognosis, and treatment planning. These slides reveal crucial information such as the presence, type, degree of differentiation, and mitotic activity of tumor cells, as well as the size, growth, and necrosis of tumor tissue when examined under a high-magnification microscope. Histopathological examination is widely regarded as the gold-standard for cancer diagnosis and subtype determination. Although manually curated histopathological features from pathologists' inspection can improve prognosis prediction accuracy,^1^^,^^2^ the process is time-consuming, subjective,^3^^,^^4^ and highly specialized, hindering the widespread use of histopathological features in clinical applications, particularly in developing countries or rural settings.^5^

Whole-slide images (WSIs) are digitized versions of conventional slides, making it easier to store and share histopathological information. The increasing popularity of WSIs also facilitates the development of computational pathology solutions that focus on the automatic interpretation of histopathological images using computational approaches, such as deep learning.^6^^,^^7^ One group of computational pathology tasks is segmenting tumor regions on a WSI.^8^, ^9^, ^10^, ^11^ Another group of tasks involves providing whole slide-level inferences, such as providing diagnosis and prognosis information.^12^, ^13^, ^14^, ^15^, ^16^, ^17^, ^18^ This study will focus on the second group of tasks.

As a result of the remarkable success of convolutional neural network (CNN)-based models, such as Residual Network (ResNet), in the image analysis realm,^19^ a large number of studies have adopted these models for automatic feature extraction from WSIs to inform patient diagnosis and prognosis.^20^^,^^21^ However, feeding the entire WSI into a machine learning model is not feasible due to WSI size and current hardware capacities.^22^^,^^23^ A typical WSI can be extremely large, containing billions of pixels, which is several orders of magnitude larger compared to natural images, making it impossible to build a CNN with an appropriate receptive field while maintaining manageable GPU memory usage. Alternatively, a multi-step method is usually adopted that analyzes a WSI in smaller portions instead of all at once.^20^^,^^24^ The first step in these pipelines normally involves dividing the WSIs into smaller units known as patches, typically with a size of several hundred pixels (e.g., 224×224). A pre-trained feature extractor is then employed to transform these patches into feature vectors. The next step involves utilizing an aggregation method to derive the overall representation of the WSI from the feature vectors of several sampled patches. Finally, this global representation vector is used to make predictions at the slide level.

However, one significant drawback of the multiple instance learning (MIL)-based aggregation methods is that they do not consider the spatial arrangements and associations of the patches and their patterns, which can limit the recognition and integration of features beyond a single patch. Although the recently emerged Vision Transformer (ViT) has the capacity to encode positional information,^25^^,^^26^ its effectiveness is hindered in digital pathological settings by the considerable number of parameters and the relatively limited availability of labeled datasets.

To address this challenge, we develop a method named Masked Pre-training for Histology Images using Transformer (MaskHIT), as a pre-training and fine-tuning pipeline for whole-slide level representation and analysis. Our proposed methodology can effectively integrate both low- and high-level features from a WSI with a hybrid approach, with the low-level features extracted using pre-trained CNN models from tissue patches, whereas the high-level features represented with ViT model that facilitates learning the relationships among patches. The self-attention mechanism and positional encoding capabilities of the transformer model allow for the integration of information spanning beyond a single patch. On the whole-slide level, we adopted the MIL method to aggregate region-level features extracted from ViT to reduce computational cost.

To further enhance the performance of the transformer model, we undertake a pre-training step using a subset of The Cancer Genome Atlas (TCGA) database through the masked patch prediction method. This inpainting procedure involves randomly obscuring a portion of the patches and training the model to reconstruct the masked patches. While fine-tuning for downstream tasks, the predictions generated by the model can also be explained by highlighting the regions of the WSI that have the most significant impact on the prediction. These indicative patches can be visualized and reviewed by clinicians to facilitate the recognition of the associations between various morphological attributes and the slide-level labels in research and clinical practice.

Our main contributions are summarized below: (1) We extended the conventional hybrid representation of WSIs by the introduction of transformer layers to take advantage of the positional relationships among patches which were neglected by previous MIL-based approaches. (2) We developed a contrastive learning-based self-supervised learning task to restore randomly masked patch features. We pre-trained the transformer model on large WSI datasets without using their labels to learn the underlying patterns of histology images. (3) We extensively evaluated our methods against popular MIL-based and recently emerged transformer models on broad groups of tasks, including survival prediction, and subtype/grade classification. MaskHIT achieved improved performance compared to the state-of-the-art MIL and transformer models. (4) We designed an adaptation attention visualization pipeline to localize high attention region on the whole slide, which agrees with pathologist's annotation of region of interest (ROI).

## Related work

### Multiple instance learning for WSIs

The unique challenge posed by WSIs is the need to integrate high-level features from numerous smaller images. Because labels are typically only available at the slide- or patient level, this aggregation is considered a form of weakly supervised learning. The most widely employed approach in this field is MIL, which treats a WSI as a bag of patches and neglects the positional information and relationships among those patches. For example, the maximum value from patch predictions was used to make slide-level classification predictions, assuming the presence of at least one positive instance when the slide is positive.^27^ On the other hand, for prognosis prediction tasks, average pooling over patch features is commonly used.^20^^,^^28^ However, these fixed operations lack the required adaptability for different domains.

Extensions of the MIL approach utilize the attention mechanism, which assigns weights to patches in the aggregation process based on their corresponding visual patterns. Ilse et al. implemented a flexible module that assigns learnable weights to each instance and aggregates patch features dynamically through a weighted average approach.^29^ Similar attention mechanism is also used in slide-level classification tasks. For example, a 3D convolutional filter was used to compute the attention score of each patch for patch feature aggregation.^10^ In another study, a clustering loss was employed to impose constraints on the feature space identified by the attention module for slide-level classification tasks.^14^

The attention-based approach is used for predicting survival outcomes as well. Yao et al. utilized a multi-cluster approach, where each patch was categorized into one of several clusters and an attention layer was employed to integrate features from each cluster^13^ Previously, our team introduced a multiple-head attention approach, which enables multiple attention layers to work in collaboration to achieve a comprehensive feature extraction.^30^

### Vision transformer in computational pathology

The transformer model was introduced in the realm of language modeling and specifically utilized for sequence-to-sequence learning tasks such as machine translation.^25^ One of the defining features of the transformer model is the incorporation of the self-attention mechanism, which allows each element within a sequence, such as a word within a sentence, to consider information from all other elements in the sequence and determine their contributions to the encoding of the current element. Additionally, positional encoding is utilized to capture the relative position of each element within the sequence. Furthermore, the recently introduced ViT model has demonstrated exceptional performance in image classification tasks without relying on CNNs.^26^ Given the vast number of parameters involved in this model, various pretext tasks were devised to train the model in an unsupervised manner. For example, the BERT language model was developed to leverage the transformer architecture and utilized masked word prediction and next sentence prediction tasks for training purposes. This approach yielded remarkable results and new advances in natural language processing.^31^

The transformer model has been introduced to the digital pathology field as well. For example, multiple studies have used the transformer model as an aggregation method under the framework of MIL for whole slide-level prediction tasks.^32^, ^33^, ^34^ Transformer model has also been pre-trained using a contrastive learning approach to replace ResNet as the backend for patch-level feature extraction.^35^ In addition, Chen et al. implemented hierarchical transformer modules (HIPT) that can be pre-trained to learn high-resolution representations of WSIs.^33^ HIPT pre-training adopted the DINO framework^36^ with a student and a teacher network, and the model is trained so that the probability distribution of the output from the student network can match that from the teacher network. However, given that the DINO pre-training framework operates on the scale of whole slides, it is more susceptible to the limited amount of WSIs. Our work mitigates this issue by predicting randomly masked patches, which are magnitudes larger in quantity, thus providing more variation to the contrastive learning procedure.

## Methods

### Model architecture

**Fig. 1:**
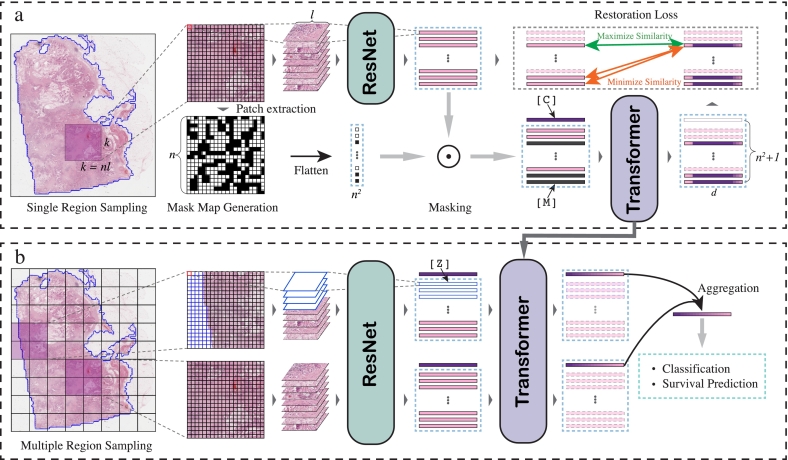
MaskHIT Architecture. (a) Pre-training stage. (b) Fine-tuning stage. [M]: mask token. [C]: class token. [Z]: zero padding.

An overview of our pipeline is shown in Fig. 1. We first randomly choose a region from WSI and break it down into non-overlapping patches. A pre-trained ResNet model serves as a fixed feature extractor for patch processing, converting each patch into a feature vector. Meanwhile, a random mask is generated for the region and is used to mask out the preprocessed feature matrix. This masked input is then sent to the transformer model along with positional information. The output from the transformer model is used to restore the original unmasked patch features. Following the pre-training step, regions are sampled from the WSI randomly or through systematic sampling procedures, and no masking is performed at this stage. Those regions are then processed via the pre-trained transformer model, and their output class tokens are aggregated for downstream tasks.

### Hybrid representation of whole-slide image

In accordance with MIL framework for WSIs, a WSI, X, can be viewed as a bag of *patches*:

$$X=\left\{P_{1},P_{2},\ldots ,P_{N}\right\}$$

where $P_{i}$ refers to one patch with size l×l (e.g., 224×224) pixels selected from the WSI and N is the total number of patches from this WSI. However, as this approach treats the patches as independent, their spatial relationships are ignored during modeling. We sought to address this problem by representing the whole slide as M large *regions* of size nl×nl (e.g., 4480×4480 when n=20 and l=224) pixels that contain high-level structure of a tissue.

$$X=\left\{R_{1},R_{2},\ldots ,R_{M}\right\}$$

and

$$R_{i}=G\left(\left(P_{i,1},x_{i,1},y_{i,1}\right),\left(P_{i,2},x_{i,2},y_{i,2}\right),\ldots ,\left(P_{i,n^{2}},x_{i,n^{2}},y_{i,n^{2}}\right)\right)$$

where $x_{i,j},y_{i,j}$ is the position of the jth patch on region i. We choose G to be the vision transformer model as it can effectively model the positions of patches.

In a typical vision transformer model, an image with a regular size is first split into small patches.^26^ The patches are flattened into a column vector, positionally encoded, and then fed to a multiple-layer transformer model together with a special token (*class* token) to capture the global information of the image. This process naturally fits the multi-stage framework of modeling WSIs. Similarly, we can sample a large “image” $X^{k}$ from WSI, then apply the pre-trained ResNet model for patch feature extraction and the transformer model for patch feature aggregation. This hybrid approach combining a CNN model with a transformer encoder has been shown to deliver improved performance for datasets with small-to-moderate sample sizes,^26^ and can also help us focus on the training of the transformer part of the pipeline.

For each patch, we first add the feature vector with a positional vector.^25^^,^^37^ For the feature representation of the patches with a dimension of d, we randomly initialize two learnable positional matrices $P_{x},P_{y}\in {\mathbb{R}}^{n\times \left(d/2\right)}$ for x- and y-axis locations. At position $\left(x_{j},y_{j}\right)$, the positional representation of the patch is the concatenation of x- and y-axis positional encodings: $\mathrm{concat}\left(P_{x}\left[x_{j}\right],P_{y}\left[y_{j}\right]\right)$. A learnable class token is appended to the start of transformer's input sequence. This token serves as the high-level representation of a WSI during training and can be used as the region-level feature vector for downstream tasks.

This sequence is inputted to an L-layer transformer encoder with each layer containing an H-head self-attention module.^25^ Patches that are not from the foreground regions are zero-padded. We apply masked multi-head self-attention in the transformer model to zero out the padded patches.

### Pre-training with masked patch prediction

Given the large number of parameters in the multi-layer transformer model, and the relatively small size of labeled WSI datasets, training the transformer model directly for outcome prediction would lead to serious overfitting problems during optimizing a transformer model. Therefore, we first pre-train the transformer model in an unsupervised manner before optimizing it for downstream tasks. This approach can greatly benefit from publicly available, unlabeled datasets. Our masked patch prediction method for pre-training operates as follows.

First, we apply a random masking procedure on patches with a fixed probability of p utilizing the blockwise masking technique.^38^ The technique involves masking multiple patches at a time until the desired masking rate of p is achieved. The feature vectors of the masked patches are then substituted with a learnable *mask* token. The positional encoded patch feature sequence and masked patches are fed into the transformer model. The restoration loss is calculated based on the output from the last layer of the transformer model at the masked locations. The unmasked patches and background patches are excluded from the pretext loss calculation. All selected patches from a batch are mixed in a single list of length k, regardless of their region or slide membership. Here, $y_{i}\in {\mathbb{R}}^{d}$ refers to the ith output vector from the transformer model, and $x_{i}\in {\mathbb{R}}^{d}$ refers to the corresponding unmasked feature vector of this patch.

The function consists of two essential components. The first component aims to maximize the similarity between the output features of the masked patches and their original ResNet representations. This is achieved by calculating the L2 distance between the output from the final layer and the original unmasked feature representation. The second component is a modified version of the InfoNCE loss, which can minimize the similarity between an output instance and all other selected input feature sequences.^39^^,^^40^ For ith entry in the mixed output, the loss is expressed as:

$$L_{i}=\alpha ∥x_{i}-y_{i}{∥}_{2}+\mathrm{\beta l}\left(x_{i},y_{i}\right)$$

where

$$l\left(x_{i},y_{i}\right)=-\log\frac{\exp\left(∥x_{i}-y_{i}{∥}_{2}/\tau \right)}{\Sigma_{j=1}^{k}\exp\left(∥x_{j}-y_{i}{∥}_{2}/\tau \right)}$$

α and β values determine the relative contribution of each component, and they are selected through a grid search method (Effect of restoration loss function Section). The temperature parameter τ is set to be 0.1 following a previous study.^41^

### Fine-tuning for whole-slide level predictions

After pre-training the transformer model using large unlabeled datasets, we can fine-tune the model for the specific tasks of interest with datasets not used in the pre-training. To accomplish this, we select a fixed number of regions from the WSI and feed them to the pre-trained transformer model without undergoing patch masking. Upon obtaining the class tokens from each region, a straightforward aggregation method is employed to calculate an average of these class tokens, which serves as the representation vector for the entire slide. This average class token can then be utilized to make slide-level predictions. A lower learning rate is assigned to the transformer component of the model to allow gradual gradient-based updates for downstream tasks.

For the downstream prediction tasks, more regions are sampled from a WSI during each iteration to ensure comprehensive coverage. The maximum overlap between any two regions is set to 50% to avoid extensive repeated sampling. The learning rate for the baseline methods is $1\times 10^{-3}$ for the survival prediction tasks and $3\times 10^{-3}$ for the classification tasks. While fine-tuning the MaskHIT model, we used the same learning rate (i.e., $1\times 10^{-3}$ or $3\times 10^{-3}$) for the last fully connected layer as the baseline methods and a small learning rate (i.e., $1\times 10^{-5}$) for the transformer backend. To prevent overfitting, an early stopping approach is utilized, with the training process ending after five stagnant epochs.

Our downstream predictions include two different types of tasks: cancer survival prediction and cancer classification. The survival outcome is the time (in years) from cancer diagnosis to the recorded death. We used the negative log Cox Partial Likelihood Loss,^42^ which is defined as:

$$l\left(\theta \right)=-\frac{1}{N_{\delta =1}}\Sigma_{i:\delta_{i}=1}\left(\hat{h_{\theta }}\left(x_{i}\right)-\log\Sigma_{j\in R\left(T_{i}\right)}e^{\hat{h_{\theta }}\left(x_{j}\right)}\right)$$

where $\hat{h_{\theta }}\left(x_{i}\right)$ is the output (i.e., risk score) of a model with parameters θ and input $x_{i}$, δ=1 means having the event (death), and $R\left(T_{i}\right)$ is patient $i^{’}s$ risk set (i.e., having not observed the event at time $T_{i}$). The cross-entropy loss was used in the classification tasks and the model performance was evaluated using the macro-averaged AUC score.

## Experiments

We have conducted two series of experiments to evaluate MaskHIT. In the first series of experiments, we pre-trained the MaskHIT model with ResNet-18 features at 10× magnification (MaskHIT-10×) using five cancer datasets from TCGA, and then compared it on downstream cancer survival prediction (*Survival-1*) and classification (*Classification-1*) tasks to other MIL-based approaches. We also conducted ablation experiments to verify the model design choices. This series of experiments aims to prove ViT's superiority over MIL-based approaches as they are agnostic of positional information. The choice of smaller dataset for pre-training is to accelerate the selection of different contrastive learning methods. In the second series of experiments, we pre-trained the MaskHIT model with ResNet-34 features at 20× magnification (MaskHIT-20×). We selected 14 cancer datasets from TCGA in this pre-training. Then we compared our model to the latest transformer-based approaches on a different set of tasks (*Survival-2* and *Classification-2*).

### Datasets

Table 1 summarizes the information about the datasets that we used in our experiments. Additional information regarding each dataset is available in Supplementary Table S1. And the detailed usage of datasets for each experiment is shown in Supplementary Table S2. Our experiments were limited to utilizing only Formalin-Fixed Paraffin-Embedded (FFPE) slides to avoid the excess artifacts on frozen slides.

**Table 1:** Summary of datasets and usage in experiments.

	Experiments	Tasks	Datasets	Slides	Regions	Patches ($\times 10^{6}$)
PT	MaskHIT-10×	1	5	2244	35,509	19.810
	MaskHIT-20×	1	14	7095	99,134	60.686
FT	Survival-1	5	5	3065	39,408	26.880
	Classification-1	3	4	3308	41,140	26.481
	Survival-2	6	7	3513	47,091	31.344
	Classification-2	3	6	3108	42,003	25.781

At 10× region size is 4480×4480 pixels and patch size is 224×224 pixels. At 20× region size is 8960×8960 pixels and patch size is 448×448 pixels.

Pre-training of the MaskHIT-10× model utilized five cancer datasets. These datasets consist of 2244 slides and about 20 million patches. Pre-training of the MaskHIT-20× model was extended to 14 cancer types from TCGA which were about 2 times more data than MaskHIT-10× model, as indicated in Table 1.

A summary of downstream fine-tuning experiments is presented in Table 2. Survival-1 tasks utilized five cancer datasets from TCGA that were not included in training the MaskHIT-10× model, which are breast cancer (BRCA), colon adenocarcinoma (COAD), brain lower grade glioma (LGG), lung adenocarcinoma (LUAD), and ovarian serous cystadenocarcinoma (OV). The three tasks of classification-1 are: (1) glioblastoma multiforme (GBM) versus LGG classification; (2) BRCA molecular subtypes classification, namely, HER2-enriched, luminal A, luminal B, and basal-like; and (3) renal cell carcinoma (RCC) subtype classification, which are clear cell, papillary, chromophobe, benign, and oncocytoma. The final task used one internal dataset from Dartmouth Hitchcock Medical Center (DHMC), whereas all other tasks used TCGA datasets.

**Table 2:** Summary of downstream experiments.

	Series 1: Comparing to MIL-based methods	Series 2: Comparing to transformer-based methods
WSI-resolution	10×	20×
Baseline methods	**MIL-AP:** MIL average pooling **MIL-Attn:** MIL attention **DeepAttnMISL:** Deep attention multiple instance survival learning **MHAttn:** Multi-head attention	**H2T:** Handcrafted histological transformer **HIPT:** Hierarchical image pyramid transformer **TransMIL:** Transformer-based correlated multiple instance learning
Survival tasks	BRCA, COAD, LGG, LUAD, OV	IDC, CRC, CCRCC, PRCC, LUAD, STAD
Classification tasks	**DHMC-RCC:** Clear cell, papillary, chromophobe, benign, and oncocytoma **TCGA-Brain:** LGG vs. GBM **TCGA-BRCA:** HER2-enriched vs. luminal A vs. luminal B vs. basal-like	**Breast:** Invasive ductal vs. invasive lobular **Kidney:** KIRC vs. KIRP vs. KICH **Lung:** LUAD vs. LUSC

Survival-2 and Classification-2 tasks followed the setup in HIPT^33^ for equitable comparison. There are six survival tasks, namely clear cell renal cell carcinoma (CCRCC), stomach adenocarcinoma (STAD), invasive ductal carcinoma (IDC), colorectal cancer (CRC), LUAD, and papillary renal cell carcinoma (PRCC). Moreover, there are three classification tasks, namely, breast (invasive ductal vs. invasive lobular), lung (adenocarcinoma vs. squamous cell carcinoma), and kidney (clear cell vs. papillary vs. chromophobe).

The tissue mask of WSIs was calculated at a low magnification level (32 μm per pixel, or 0.3125×) using the purple thresholding method for thumbnail images and further processed with binary dilation and erosion to eliminate small holes and debris. We then sequentially extracted patches at 10× (1 μm per pixel) and 20× (0.5 μm per pixel) from WSIs without overlap. At least 5% of pixels within a patch had to be from tissue for that patch to be considered foreground. ResNet-18 (or ResNet-34 for 20×) model pre-trained using the ImageNet was used as the fixed feature extractor to process all the foreground patches. The extracted feature vectors, with a dimension of 512, were saved on storage before the experiments to cut on computational cost during model training and testing. Background patches were excluded from the feature extraction step and padded with zero vectors. Also, the position of each patch within the region $\left(x,y\right)$ was recorded.

At 10× region size is 4480×4480 pixels and patch size is 224×224 pixels. At 20× region size is 8960×8960 pixels and patch size is 448×448 pixels.

### Implementation details

#### Masked pre-training

At 10× magnification, we set the region size to be 4480×4480 pixels in our experiments so each region contains 400 non-overlapping 224×224-pixel patches. This region size is selected to balance computational feasibility and the ability to identify high-level morphological structures. The computational cost of the transformer model increases quadratically with the input sequence length, hence, a larger region would be computationally unfeasible, whereas a smaller region may not be capable of capturing significant histological information. While choosing a region from WSI, we require at least 25% of its patches to be foreground patches. Regions that are primarily background may only provide limited information, whereas selecting only regions that do not overlap with the background would greatly reduce the number of regions available for sampling. The extracted features of the foreground patches in a region and the zero paddings for the background patches as well as their corresponding locations are sent to the transformer model. We randomly sampled two regions from every WSI in each iteration.

We follow the implementation of ViT^26^ and set L=12 and H=8, as the number of layers and attention heads, respectively, in our experiments. This model is similar to ViT-Base but with fewer heads (8 vs. 12) to accommodate the output dimension of the ResNet model (d=512).

The learning rate is $4\times 10^{-5}$, and is warmed up with the first 8000 steps and then annealed according to the cosine schedule. For pre-training, 80% of the data are for optimizing the transformer model, whereas the remaining 20% are used to monitor the loss. With a batch size of 64, the model was pre-trained for a total of 400,000 iterations using the AdamW optimizer.^43^ Each training step took approximately 1.07 s with two V100 GPUs.

#### Comparison with other methods

In the first series of evaluations, we used 5-fold cross-validation for model evaluation. During each fold, 20% of the data was set aside for testing purposes, 20% was used for performance monitoring, and the remaining 60% was utilized for model training. Specifically, for the survival prediction tasks, given the limited number of events, we noted a substantial variation in survival prediction performance depending on model initialization. As a result, we repeated the 5-fold cross-validation process five times and presented the average test c-index for a more stable evaluation.

In the second series, we compared MaskHIT to three other transformer-based approaches, HIPT,^33^ TransMIL,^32^ and H2T.^44^ The HIPT study is built upon transformer modules and pre-trained with large datasets. The H2T approach is a lightweight representation framework utilizing the co-localization of histological patterns. The TransMIL method introduces a TPT module comprising two transformer layers for aggregating morphological information and a pyramid position encoding generator for encoding spatial information. Given the different data preprocessing pipelines used in the HIPT study, we used the HIPT results directly from the corresponding publication. To ensure fair and consistent comparison, we used the same data split as released in the HIPT repository to evaluate TransMIL, H2T, and MaskHIT.

## Results

### Self-supervised pre-training

**Fig. 2:**
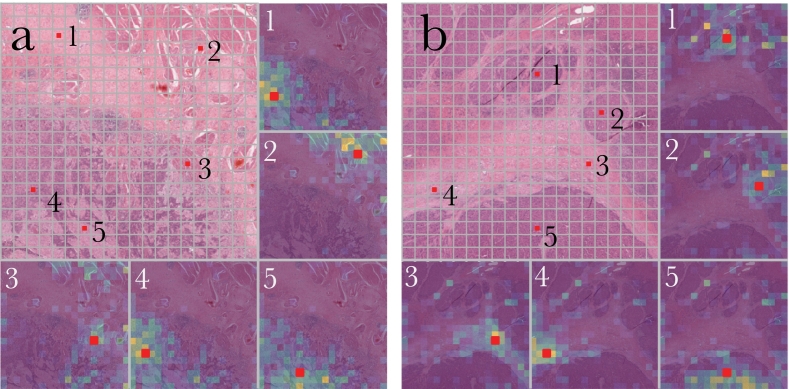
Attention map visualization for the pre-trained model. (a) BLCA. (b) LIHC. Patches with a red dot are the query patches. The intensity of the color indicates the extent of attention that the query patches pays to each patch, with brighter colors representing a higher score and darker colors representing a lower score. (For interpretation of the references to color in this figure legend, the reader is referred to the web version of this article.)

In the self-supervised pre-training stage, we randomly mask a portion of patches from each region and let the transformer model restore those masked patches so it can learn the underlying structure of WSI. After pre-training, we visualized the attention map learned by the MaskHIT model as a qualitative evaluation (Fig. 2). Specifically, we chose several patches from one region on WSI as queries and calculated attention weights from other patches (as keys). These weights serve as an indicator of the extent to which the query patch relies on information from other patches. Our observations indicate that each patch tends to draw information from similar patches and those located in close proximity. These observations support our objective to train a transformer model that can learn spatial relationships between patches in histopathological images.

### Downstream prediction

#### Comparison with MIL-based approaches

Survival-1 results are summarized in Table 3. In this series of evaluations, we compared MaskHIT to several state-of-the-art (SOTA) MIL approaches. The first baseline method uses average pooling to aggregate feature vectors from patches (referred to as MIL-AP).^20^^,^^28^ We also included three attention-based MIL methods, namely MIL-Attn,^29^ DeepAttnMISL,^13^ and MHAttnSurv.^30^ We kept the data sampling approaches exactly the same across these different methods, to demonstrate the effectiveness of MaskHIT for feature aggregation. All experiments were conducted at 10× magnification. Results show that MaskHIT consistently outperforms or matches the baseline methods across different cancer types in terms of average cross-validation c-index. On average, across the five cancer types, MaskHIT achieves a c-index of 0.612 with a standard deviation of 0.004 across repeated cross-validation, which is 3.0% higher than the best-performing baseline MHAttn (0.594) and the difference is statistically significant (*p*=0.008).

**Table 3:** Survival prediction performance, average c-index ± standard deviations.

Architecture	BRCA	COAD	LGG	LUAD	OV	Avg
MIL-AP^20^	0.579 ± 0.010	0.600 ± 0.017	0.643 ± 0.019	0.551 ± 0.007	0.568 ± 0.021	0.588 ± 0.005
MIL-Attn^29^	0.566 ± 0.025	0.583 ± 0.014	0.655 ± 0.007	0.560 ± 0.014	0.587 ± 0.016	0.590 ± 0.004
DeepAttnMISL^13^	0.575 ± 0.012	0.580 ± 0.011	0.634 ± 0.018	**0.561** **±** **0.014**	0.596 ± 0.016	0.589 ± 0.006
MHATTN^30^	0.579 ± 0.020	0.592 ± 0.025	0.657 ± 0.007	0.554 ± 0.012	0.588 ± 0.009	0.594 ± 0.008
MaskHIT	**0.602** **±** **0.012**	**0.608** **±** **0.022**	**0.685** **±** **0.009**	**0.561** **±** **0.009**	**0.602** **±** **0.008**	**0.612** **±** **0.004**

Classification-1 results are summarized in Table 4, which shows MaskHIT outperforms all the baseline approaches on all three classification tasks by a significant margin. MaskHIT achieves an AUC of 0.958, 0.964, and 0.735, for DHMC-RCC, TCGA-brain and TCGA-BRCA, respectively. The AUC improvement of MaskHIT over the best-performing baseline (MHAttn) is 2.1%, 1.9%, and 2.7% for the three tasks. The average improvement is statistically significant (*p*=0.001).

**Table 4:** Cancer subtype classification performance, average AUC ± standard deviations.

Architecture	DHMC-RCC	TCGA-Brain	TCGA-BRCA
MIL-AP^20^	0.909 ± 0.014	0.919 ± 0.032	0.689 ± 0.048
MIL-Attn^29^	0.932 ± 0.022	0.925 ± 0.026	0.693 ± 0.050
DeepAttnMISL^13^	0.911 ± 0.041	0.928 ± 0.031	0.667 ± 0.034
MHATTN^30^	0.938 ± 0.028	0.946 ± 0.027	0.716 ± 0.042
MaskHIT	**0.958** **±** **0.017**	**0.964** **±** **0.013**	**0.735** **±** **0.026**

#### Comparison with transformer-based approaches

Table 5 summarizes the comparison of the MaskHIT approach to the HIPT method in survival prediction tasks. H2T was excluded from this experiment as it was not evaluated for survival tasks in the original study. We find that MaskHIT-10× and MaskHIT-20× outperform HIPT on all cancer types evaluated in terms of average validation AUC with an average increase in c-index of 0.046 (*p*=0.015) and 0.071 (*p*=0.020), respectively.

**Table 5:** Cancer survival prediction performance compared with other transformer-based approaches. * Results from the original study.

Architecture	IDC	CRC	CCRCC	PRCC	LUAD	STAD
HIPT^33^*	0.634 ± 0.050	0.608 ± 0.088	0.642 ± 0.028	0.670 ± 0.065	0.538 ± 0.044	0.570 ± 0.081
MaskHIT-10×	0.642 ± 0.029	0.684 ± 0.083	0.649 ± 0.049	0.743 ± 0.081	0.599 ± 0.027	0.621 ± 0.045
MaskHIT-20×	**0.645** **±** **0.031**	**0.697** **±** **0.101**	**0.665** **±** **0.072**	**0.823** **±** **0.082**	**0.615** **±** **0.027**	**0.640** **±** **0.027**

Table 6 summarizes MaskHIT's performance compared to H2T, HIPT, and TransMIL on breast cancer, kidney cancer, and lung cancer classification tasks using 25% and 100% of available data. With 100% training, MaskHIT-20× performs better than other methods in breast and lung classification tasks. While in kidney classification task, MaskHIT-20×’s performance is similar as TransMIL (0.990 vs. 0.989). MaskHIT-10×’s performance is close to MaskHIT-20×’s for breast and kidney datasets, but 1.7% lower than MaskHIT-20× in lung cancer classification task. When 25% training data are used, MaskHIT-10× becomes the best performing model for the breast dataset, and TransMIL slightly outperforms MaskHIT-20× for the kidney dataset (0.980 vs. 0.973). MaskHIT-20× remains the best performing model for the lung cancer classification task. On average, MaskHIT-20× achieves an 8.5%, 1.7%, and 4.5% increase in AUC compared to H2T, HIPT, and TransMIL, respectively, with 25% training. When trained with all available data, MaskHIT achieves an average increase of 4.9%, 2.7%, and 3.9%, respectively. Specifically, when comparing with the overall best performing baseline model HIPT using 100% training data, the AUC improvements are statistically significant for breast and kidney cancer (*p*=0.001 and 0.029).

**Table 6:** Cancer subtype classification performance compared with other transformer-based approaches. * Results from the original study.

Architecture	Breast		Kidney		Lung	
	25% Training	100% Training	25% Training	100% Training	25% Training	100% Training
H2T^44^	0.750 ± 0.038	0.860 ± 0.060	0.956 ± 0.014	0.979 ± 0.009	0.843 ± 0.035	0.910 ± 0.030
HIPT^33^*	0.821 ± 0.069	0.874 ± 0.060	0.974 ± 0.012	0.980 ± 0.013	0.923 ± 0.020	0.952 ± 0.021
TransMIL^32^	0.783 ± 0.064	0.861 ± 0.054	**0.980** **±** **0.008**	0.989 ± 0.005	0.884 ± 0.027	0.926 ± 0.018
MaskHIT-10×	**0.867** **±** **0.042**	0.931 ± 0.047	0.972 ± 0.012	0.986 ± 0.008	0.894 ± 0.030	0.945 ± 0.026
MaskHIT-20×	0.863 ± 0.037	**0.932** **±** **0.045**	0.973 ± 0.012	**0.990** **±** **0.024**	**0.929** **±** **0.026**	**0.961** **±** **0.006**

### Ablation studies

We conducted additional investigations to determine the effects of pretext training, restoration loss function choice, number of sampled regions, and region size on downstream tasks.

#### Effect of pre-training

**Fig. 3:**
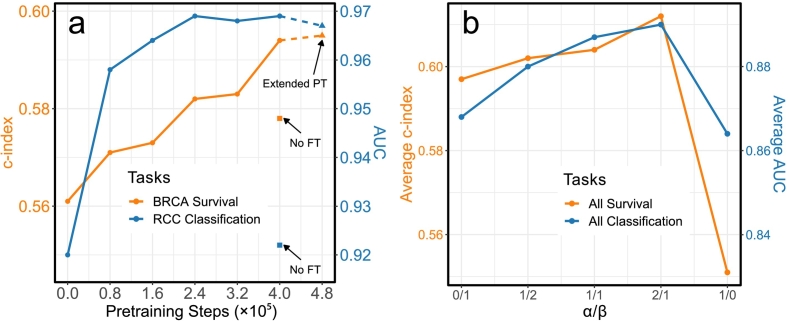
Effect of pre-training and choice of loss function components on downstream tasks. (a) Effect of pre-training. NO FT: no fine tuning - freeze transformer part when fine-tuning. Extended PT: extended pre-training - pre-train the final model for additional steps and then fine-tune. (b) Effect of the restoration loss composition. α: the coefficient of the L2 distance component; β: the coefficient of the InfoNCE loss component.

Using TCGA-BRCA as an example of the survival task, and DHMC-RCC as an example of the classification task, we show the effect of pre-training on downstream task performance (Fig. 3a). When the transformer model has not undergone any pre-training, it performs poorly on slide-level tasks; in fact, its performance can be worse than the baseline methods. Specifically, MIL-AP has a c-index of 0.579 in TCGA-BRCA survival prediction, whereas the raw transformer model only has a c-index of 0.561. This supports our intuition that using a transformer model without pre-training, given the number of parameters and the limited amount of labeled data, does not necessarily result in improved performance.

Our findings demonstrate that with a moderate level of pretext training, transformer model performance improves. Our downstream tasks are performed after the vision transformer model has been pre-trained for a total of $4\times 10^{5}$ steps. However, if we further pre-train this model for another 80,000 steps (as indicated by extended PT, short for extended pre-training in Fig. 3a), it does not lead to any additional improvements in slide-level performance, suggesting that benefits of pre-training plateau after a sufficient amount of pre-training. Additionally, Fig. 3a reveals that if the transformer component is frozen for the downstream tasks (as indicated by No FT, short for no fine-tuning in Fig. 3a), there is a significant decline in performance, which underscores the importance of using a small learning rate for the transformer model while fine-tuning for slide-level predictions.

#### Effect of restoration loss function

As our restoration loss consists of two components, L2 distance with weight α and InfoNCE loss with weight β, we evaluated how different combinations of these components could affect the downstream task performance. We reported the average c-index for the five survival prediction tasks and the average AUC for the three cancer subtype classification tasks in series 1, with the results summarized in Fig. 3b.

Fig. 3b suggests that integrating both L2 distance and InfoNCE loss yields better downstream performance compared to employing either one in isolation, with optimal downstream performance attained when setting α=2 and β=1. InfoNCE loss encourages differentiable representations of the restored patches, fostering smooth transitions in the learned features. In contrast, L2 distance emphasizes the similarity between the features of the restored patches and their originals. The synergistic effect of combining both losses is evident, enabling the restored patch features to be both differentiable and closely aligned with their original counterparts, striking a balance that enhances the overall efficacy of the model.

#### Effect of the number of patches and the number of regions

**Fig. 4:**
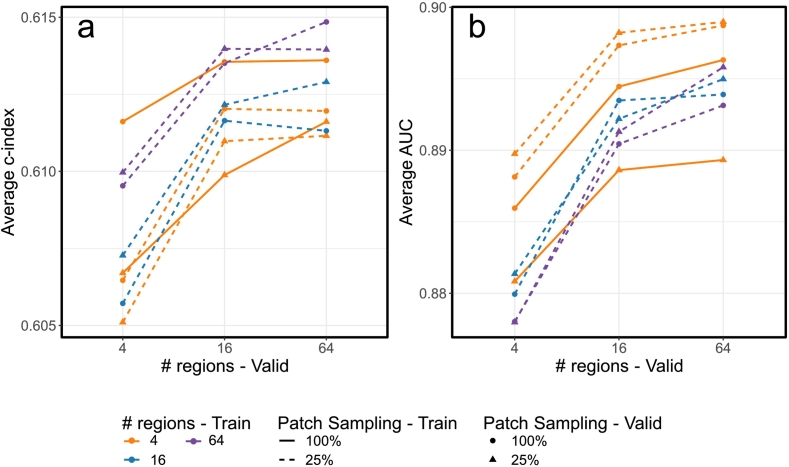
Effect of region and patch sampling at training and validation (valid) time on prediction performance. (a) Average c-index. (b) Average AUC.

We further evaluated the effects of region and patch sampling procedures on the downstream tasks. The results are presented in Fig. 4a for survival tasks and Fig. 4b for classification tasks. The solid orange lines represent models trained with four regions sampled from each WSI and 100% of patches sampled from each region. We will refer to this sampling schema as four regions with 100% coverage, or 4×100% for short. The series with round dots are models evaluated with 100% coverage, and the series with triangles are models evaluated with 25% coverage. We found that when more regions are sampled from each WSI during the evaluation time, the average test performance also increases. Additionally, sampling with 100% coverage at evaluation time outperforms 25% coverage by a large margin.

Focusing on the dashed lines, which refer to models trained with 25% coverage, we can evaluate the effect of the number of regions sampled at training time on the whole slide-level prediction performance. For the survival prediction task, we found that sampling more regions from each WSI is associated with an increased test c-index. However, the trend is the opposite for the classification tasks, as a higher number of regions sampled at training time is associated with worse test AUC. The effect of patch sampling at evaluation time is inconsistent across our experiments, but the number of regions sampled at evaluation time is mostly associated with better performance.

Among all these training/evaluation scenarios, the best test performance for the survival prediction tasks is 0.615, achieved when training the models using 64×25%, and evaluating with 64×100%. The best performing model for the classification task is trained with 4×25% and evaluated with 64×25%, with an average AUC of 0.899.

#### Effect of region size

**Fig. 5:**
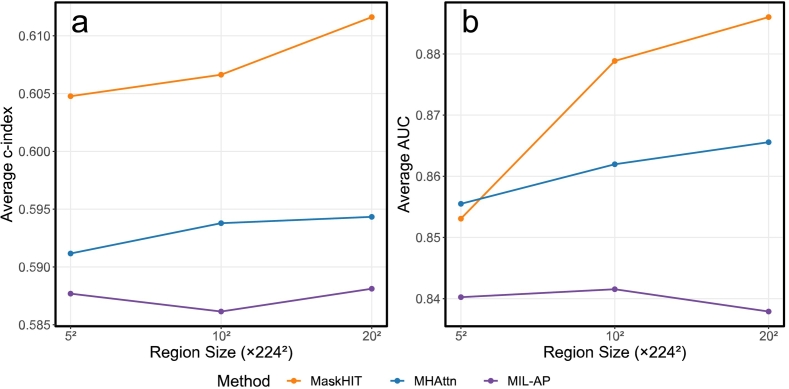
Effect of region size on MaskHIT, MHAttn, and MIL-AP on prediction performance. (a) Average c-index of survival prediction tasks. (b) Average AUC of classification tasks.

We evaluated the effect of region size on the prediction performance for survival tasks and classification tasks, and presented the results in Fig. 5a and Fig. 5b, respectively. In addition to MaskHIT, we included two additional methods as baselines: the plain MIL method, MIL-AP, and the attention-based method, MHAttn. We experimented with three region sizes: the small region with ${\left(5\times 224\right)}^{2}$ pixels, the medium region with ${\left(10\times 224\right)}^{2}$ pixels, and the large region with ${\left(20\times 224\right)}^{2}$ pixels. We trained the models using four large regions, and evaluated them with either 64 small regions, 16 medium regions, or 4 large regions, to maintain consistency in the total number of patches across experiments.

The results indicate that, for MaskHIT, larger region sizes result in better survival and classification performance, with the trend being more evident for the classification tasks. For the MHAttn method, an increase in performance is also observed when evaluating using larger regions, but the trend is mild compared to the MaskHIT model. This ablation study demonstrates that the MaskHIT model benefits from larger region sizes compared to MIL-based approaches, and a region size of 4480×4480 pixels at 10× resolution is a good candidate. Although the performance does not saturate with region size, increasing it further would quadratically increase the computational costs and was not explored. The results for the MIL-AP method are mixed without clear trend, which agrees with our expectation as this MIL-based approach does not consider positional information and therefore cannot benefit from larger region size.

### Attention map visualization

To understand MaskHIT's predictions, an analysis of the attention maps from selected WSIs was conducted. The procedure involved averaging out attention weights obtained from different attention heads for each layer, followed by applying recursive multiplication of this weight matrix across all attention layers to calculate overall attention weights. We extracted the attention weights of the class token, which served as a representation of the information incorporated in the class token from each patch.

We calculated attention maps using both pre-trained model and model fine-tuned for the downstream tasks. Their difference highlights the shift in the class token's attention from the targetless pre-training task to the guided WSI-level prediction tasks, thus providing insight into the model's attention mechanism during the prediction process.

**Fig. 6:**
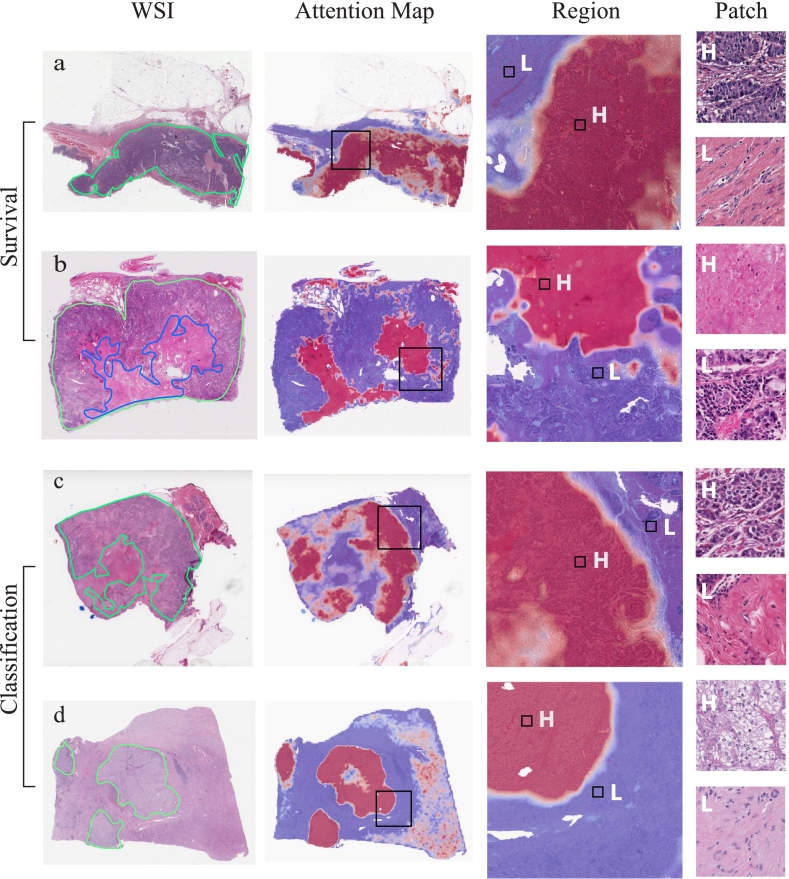
Visualization of attention maps. Region size: 4480 μm, Patch size: 224 μm. (a) COAD; (b) LUAD; (c) BRCA; (d) KIRC. Red is the color for higher attention (H), and blue is the color for lower attention (L). First column labeled with “WSI” shows pathologist's annotations of the tumor in green color and necrosis in blue color. (For interpretation of the references to color in this figure legend, the reader is referred to the web version of this article.)

Fig. 6 presents a sample from each of the COAD, LUAD, BRCA, and KIRC datasets from TCGA, with attention maps obtained using the MaskHIT-10× model for survival tasks in COAD and LUAD, and molecular subtype and cancer classification tasks in BRCA and KIRC, respectively. The COAD example shows good alignment between the higher attention areas detected by the model and the tumor region identified by the pathologist. In the LUAD example, where the tumor extends to the entire slide, the model pays more attention to regions featuring tumor necrosis, which is typically associated with favorable patient outcomes. Additionally, in the BRCA and KIRC examples, the model's higher attention areas align well with pathologist annotations of the tumor region, demonstrating the model's ability to successfully relocate attention for each specific downstream task. Additionally, we present the visualization of the same set of examples but obtained from MaskHIT-20×in Supplementary Fig. S1, and the model generates similar attention patterns as MaskHIT-10×.

## Discussion

We evaluated MaskHIT on various types of tasks, including survival prediction, subtype classification, grade classification, and molecular subtype classification. Subtype and grade classification are usually easier tasks as they are typically based on visual patterns, such as cell size and shape, mitotic rate, and the presence or absence of certain cell types. However, histopathological examination alone is not definitive for determining the molecular subtype of the tumor, which generally requires molecular or genomic analysis of the tumor tissue. Moreover, survival prediction using histological images is challenging given the lack of definitive prognostic features for most cancer types. Despite these variations, we found that MaskHIT consistently outperforms existing methods, which demonstrates the robustness of our approach for a wide spectrum of tasks.

One limitation of our approach is that we still rely on the MIL framework to represent WSIs. As demonstrated in Fig. 5, the vision transformer model can benefit from a larger region size. Although the transformer model enables us to represent a large region with 20 million pixels (or 80 million pixels at 20× resolution) from WSIs, further expanding the region size to the entire slide is not computationally feasible at this stage. Therefore, although we can learn the spatial arrangements of patches within a relatively large region, we may still lose information on the higher region-level positions. While the HIPT model was designed to solve this problem through the hierarchical use of transformer modules, pre-training the transformer model beyond the region level is still a challenging task given the limited number of slides available for pre-training.

In the future, we plan to develop additional pretext tasks and expand the collection of histopathological images to improve the representation of whole slides through pre-training. Moreover, the proposed MaskHIT model can be further enhanced through utilizing better backbone models specialized for feature extraction from medical images for patch feature representation.

Additionally, the demonstrated versatility of the MaskHIT across diverse downstream tasks suggests promising avenues for its future application. For instance, the pre-trained MaskHIT model holds potential for survival prediction or cancer subtype classification tasks in scenarios with limited sample sizes. The improved performance obtained through pre-training indicates our masked pre-training pipeline could also be applied to datasets which share different characteristics, thereby enhancing prediction performance in varied clinical settings. Furthermore, MaskHIT's capability to identify task-specific high-attention regions within WSIs has the potential to uncover novel morphological patterns or assist pathologists in more efficient slide analysis. We envision that the proposed MaskHIT model will prove effective across a spectrum of tasks and applications within computational pathology, a direction we aim to delve into in future investigations.

## Conclusion

Our study proposes a novel approach to extend the conventional multi-stage MIL-based representation of WSIs by leveraging the transformer's proficiency in learning positional associations among patches. With the integration of our unsupervised training pipeline, featuring the restoration of randomly masked patches, MaskHIT outperforms existing methods in both survival prediction and classification tasks. The visualization of self-attention maps provides compelling evidence that our pre-training task empowers the transformer model to discern spatially and morphologically relevant associations among patches. Furthermore, the adaption attention map, generated after fine-tuning the transformer model for downstream tasks, illustrates its ability to recognize clinically meaningful visual patterns. Ablation results corroborate the positive impact of pretext training and show that our transformer-based approach can benefit from using larger region sizes to capture positional information of patches. Our comprehensive findings collectively underscore the efficacy and versatility of MaskHIT in pathology image analysis.

## Code Availability

The Python implementation of the model and pre-trained weights will be available at https://github.com/BMIRDS/WSI-PLP/tree/maskhit.

## Author contributions

S.J. developed the deep learning framework, conducted the experiments, and drafted the manuscript. L.H. and A.S. served as expert pathologists for the annotation of whole slide images and the evaluation of the results. S.H. supervised the project. All authors reviewed and contributed to the manuscript.

## Declaration of generative AI and AI-assisted technologies in the writing process

The authors used AI-powered tools to proofread the manuscript. Any potential edits were solely editorial and were carefully reviewed by the authors. Otherwise, none of the presented content was generated by an AI model.

## Declaration of competing interest

The authors declare that they have no known competing financial interests or personal relationships that could have appeared to influence the work reported in this study.

## Data Availability

TCGA data can be accessed through the official TCGA data portal at https://portal.gdc.cancer.gov. The DHMC-RCC dataset is available at https://bmirds.github.io/#dartmouth-kidney-cancer-histology-dataset.
